# Bilothorax: A Case Report and Systematic Literature Review of the Rare Entity

**DOI:** 10.1155/2024/3973056

**Published:** 2024-06-21

**Authors:** Roshan Acharya, Smita Kafle, Yub Raj Sedhai, Dhan Bahadur Shrestha, Kevin Walsh, Wasif Elahi Shamsi, Suraj Gyawali, Nikita Acharya, Anthony Lukas Loschner, Edmundo Raul Rubio

**Affiliations:** ^1^ Division of Pulmonary and Critical Care Medicine Virginia Tech Carilion School of Medicine, Roanoke, VA, USA; ^2^ Department of Family Nurse Practitioner Frontier Nursing University, Versailles, KY, USA; ^3^ Division of Pulmonary and Critical Care Medicine University of Kentucky, Bowling Green, KY, USA; ^4^ Department of Internal Medicine Mount Sinai Chicago, Chicago, IL, USA; ^5^ Department of Emergency Medicine Grande International Hospital, Kathmandu, Nepal; ^6^ Department of Internal Medicine Universal College of Medical Sciences, Siddharthanagar, Nepal

## Abstract

**Background:**

Bilothorax is defined as the presence of bile in the pleural space. It is a rare condition, and diagnosis is confirmed with a pleural fluid-to-serum bilirubin ratio of >1.

**Methods:**

The PubMed, Embase, Google Scholar, and CINAHL databases were searched using predetermined Boolean parameters. The systematic literature review was done per PRISMA guidelines. Retrospective studies, case series, case reports, and conference abstracts were included. The patients with reported pleural fluid analyses were pooled for fluid parameter data analysis.

**Results:**

Of 838 articles identified through the inclusion criteria and removing 105 duplicates, 732 articles were screened with abstracts, and 285 were screened for full article review. After this, 123 studies qualified for further detailed review, and of these, 115 were pooled for data analysis. The mean pleural fluid and serum bilirubin levels were 72 mg/dL and 61 mg/dL, respectively, with a mean pleural fluid-to-serum bilirubin ratio of 3.47. In most cases, the bilothorax was reported as a subacute or remote complication of hepatobiliary surgery or procedure, and traumatic injury to the chest or abdomen was the second most common cause. Tube thoracostomy was the main treatment modality (73.83%), followed by serial thoracentesis. Fifty-two patients (51.30%) had associated bronchopleural fistulas. The mortality was considerable, with 18/115 (15.65%) reported death. Most of the patients with mortality had advanced hepatobiliary cancer and were noted to die of complications not related to bilothorax.

**Conclusion:**

Bilothorax should be suspected in patients presenting with pleural effusion following surgical manipulation of hepatobiliary structures or a traumatic injury to the chest. This review is registered with CRD42023438426.

## 1. Introduction

Bilothorax, cholethorax, or thoracobilia is defined as the presence of bilirubin in the pleural cavity. The pleural cavity has no anatomical connection to the abdominal compartment, so the presence of bilirubin in the pleural space should always be considered pathological. The pathophysiology is poorly understood and likely due to the negative pressure generated by the thoracic cavity causing bile translocation through diaphragmatic pores or defects. Bilothorax is diagnosed when the pleural fluid-to-serum bilirubin ratio is equal to or greater than one [1]. This entity appears underdiagnosed as it cannot be distinguished from other causes of hydrothorax with radiological imaging or physical examination, and its diagnosis requires a high index of suspicion. Delayed diagnosis can lead to inflammation, causing scarring and fibrosis of the pleura. We present a case of bilothorax and a review of the literature with a focus on incidence, etiology, and management. We also conducted a systematic literature review of the reported cases per the Preferred Reporting Items for Systematic Reviews and Meta-Analyses (PRISMA) guidelines.

### 1.1. Case

A 72-year-old woman with a history of atrial fibrillation, left atrial thrombus on apixaban, heart failure with preserved ejection fraction on furosemide, iron deficiency anemia (hemoglobin 9-10 mg/dL), and morbid obesity (BMI 40 kg/m^2^) underwent laparoscopic cholecystectomy for gangrenous cholecystitis a month prior to her presentation to the emergency department, reporting shortness of breath for three days. She denied fever, cough, chills, lower extremity edema, and orthopnea. The physical exam was unremarkable except for decreased breath sounds on the right lung base and a noted rapid ventricular response. A chest X-ray (CXR) revealed new-onset right-sided pleural effusion and bilateral pulmonary vascular congestion. Pro-BNP was 1815 pg/mL (it was 1120 pg/mL the month before). SARS-CoV-2 RT-PCR was positive on a nasal swab. She had leukocytosis of 16 k/*μ*L, neutrophils of 74%, hemoglobin of 10.5 g/dL, and platelets of 552 k/*μ*L. The basic metabolic profile and liver profile were grossly unremarkable. A point-of-care ultrasound revealed a moderate-sized simple pleural effusion. She was started on intravenous ceftriaxone, azithromycin, and dexamethasone. Intravenous heparin was initiated, and her apixaban was stopped for anticipated thoracentesis. She was started on 80 mg/day intravenous furosemide, and a CXR three days later demonstrated an unchanged effusion. She underwent bedside thoracentesis with the removal of 600 mL of orange-colored pleural fluid. Considering her recent cholecystectomy and pleural effusion not responsive to diuresis, we suspected a bilothorax. The pleural fluid bilirubin level was 1.4 mg/dL, and the serum bilirubin level was 0.3 mg/dL with a pleural fluid-to-serum bilirubin (PB/SB) ratio of 4.67. Pleural fluid pH was 7.21, total protein (TP) was 4.2 g/dL, lactate dehydrogenase (LDH) was 223 IU/L, white blood cell (WBC) was 1556/mm^3^, 85% lymphocytes, and glucose level was 96 mg/dL. The gram stain and culture had no growth, and cytology was negative for malignant cells. A subsequent computerized tomographic scan (CT scan) of the chest did not show diaphragm defects. A surgical consultation was obtained, and they recommended no further intervention in view of the chest CT results. After the thoracentesis, her oxygen was weaned off and her dyspnea improved. She was discharged in her usual state of health. A follow-up CXR after one month demonstrated no recurrent effusion.

## 2. Review of the Literature

### 2.1. Method

#### 2.1.1. Search Strategy

A systematic review was conducted per the PRISMA guideline (Supplementary Table 1). We searched the PubMed/MEDLINE, Google Scholar, Embase, and CINAHL databases. The Boolean parameters that were used to search were “bilothorax” OR “cholethorax” OR “biliary pleural effusion” OR “bilious pleural effusion” OR “thoracobilia”. Table 2 in the Supplementary Material section provides a detailed description of the search strategy. We included studies published before January 31, 2023. The study's protocol was registered in PROSPERO (CRD42023438426).

#### 2.1.2. Eligibility Criteria

Retrospective studies, case series, case reports, and conference abstracts were included. The comprehensive reviews were included if they had patients' information that could be pooled for description. Editorials, comments, viewpoints, and articles lacking full text were excluded. The studies in languages other than English were translated using the Google Translate website (Google LLC).

*Inclusion criteria.* The studies meeting the above eligibility criteria were included in this review if the study had patients with (1) measured bilirubin level in pleural fluid (2) and/or pleural fluid defined as green color or clearly mentioned bilious fluid/bile during thoracentesis or chest tube insertion.

*Exclusion criteria.* The studies were excluded if no pleural fluid bilirubin measurement or color description was available.

#### 2.1.3. Data Extraction

Studies were identified and screened for eligibility by two authors (R.A. and S.K.) independently based on inclusion criteria. EndNote software was used to maintain the records of identified and screened studies and remove duplicated studies. Discrepancies were resolved by mutual consent obtained from another author (D.B.S.). A Microsoft Excel sheet (Microsoft Corp.) was used to extract the study characteristics, such as type of study, year of publication, age, sex, background, pleural and serum fluid bilirubin measurements in mg/dL, pleural fluid analyses, presence of bilopleural fistula (BPF), organisms isolated, management, and outcome. The patients with reported pleural fluid and/or serum bilirubin, age, presence of BPF, and other relevant data were pooled for quantitative data analysis. If a study had multiple patients reported, then all the patients from the study were pooled.

#### 2.1.4. Outcome Measures

Our primary objective of the study was to identify the total number of reported cases of bilothorax. The secondary objectives were to calculate the ratio of pleural fluid-to-serum bilirubin and the total and differential cell counts in the pleural fluid and identify etiologies causing bilothorax, isolated organisms, radiological imaging used, treatment, and outcome.

#### 2.1.5. Statistical Analysis

The continuous variables were reported in mean with standard deviation (SD). The categorical variables were reported in frequency with percentages and 95% confidence intervals (CI). The statistical analysis was done in STATA 17.0 software (Stata Corp. LLC).

#### 2.1.6. Risk of Bias Assessment

Joanna Briggs Institute's (JBI) critical appraisal checklists for case reports, case series, and cohort studies were used for risk of bias assessment. The checklist included 8 to 11 items. If the answer to an item was yes, it was scored 1; otherwise, it was scored zero. For case reports, quality scores of 2 or less, 3 to 5, and 6 or greater were considered low, moderate, and high quality, respectively. For case series and cohort studies, quality scores of 4 or less, 5 to 7, and 8 or greater were considered low, moderate, and high quality, respectively (Supplementary Tables 3, 4, and 5).

## 3. Results

### 3.1. Literature Search

**Figure 1:**
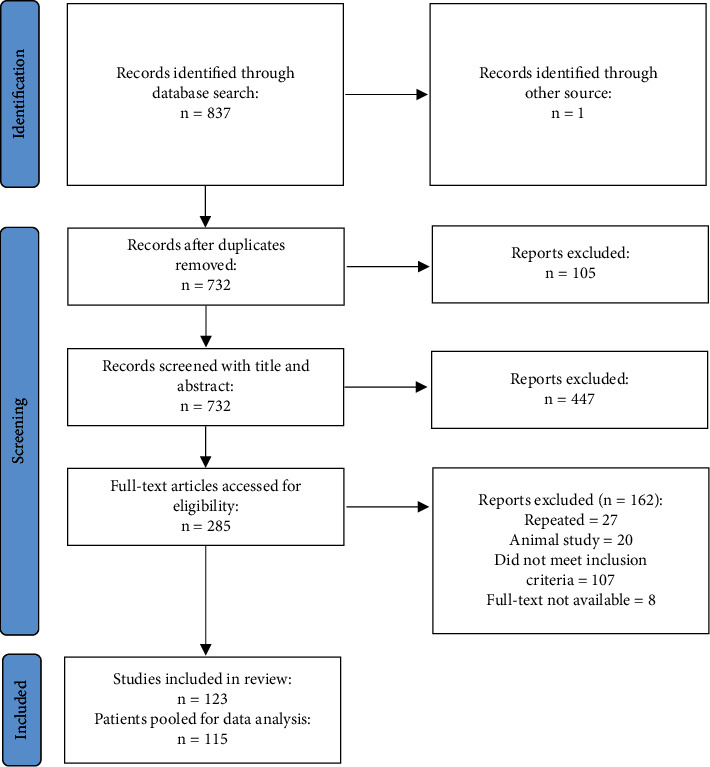
PRISMA diagram of included studies.

A total of 837 studies were identified through the databases, and one additional record was obtained from other sources. One hundred five studies were duplicated and, hence, were omitted. A total of 732 studies were screened with titles and abstracts, of which 285 qualified for full-text review. After applying inclusion and exclusion criteria, 123 studies were qualified for the qualitative analysis. From those 123 studies, 115 patients were pooled for quantitative analysis (Figure 1).

### 3.2. Primary Outcome

Of the 838 studies identified through a database search, 123 qualified for the review. Of the 123 studies, 80 were case reports, 25 were abstracts (Table 1), and 18 were observational studies (Table 2). Eighty from case reports, 25 from abstracts, and ten from observational studies—a total of 115 patients—were pooled for quantitative analysis (Tables 3 and 4).

### 3.3. Secondary Outcome

Of the 115 patients, 97 bilothorax cases were reported in the setting of surgery or surgical circumstances. The most common cause was percutaneous transhepatic biliary drainage (PTBD) which was 23.47% (*n* = 27). It was followed by trauma, gunshot, or stab injury to the right chest or abdomen at 15.65% (*n* = 18). Similarly, a liver transplant was reported in 5.21% of cases (*n* = 6) (Table 4). The most common nonsurgical etiology was cholecystitis, 5.21% (*n* = 6).

The mean pleural fluid bilirubin level was 72 mg/dL (SD 12.19), the serum bilirubin level was 61 mg/dL (SD 44), and the PB/SB was 3.47, 95% CI 2.15-4.70 (Table 4).

A bilopleural fistula (BPF) was reported in 52 patients (51.30%, 95% CI 42.13-6.039). Seventy-three types of radiological imaging were reported in 68 patients, of which 36 (52.94%, 95% CI 40.92-64.62) were able to detect BPF. CT scan was the most common imaging modality, with 51 chest CT but only a 25.92% (*n* = 14) detection rate for a diaphragmatic defect, followed by HIDA scans in 14 patients which detected a diaphragmatic defect in 78.57% of cases (*n* = 11). Magnetic resonance imaging of the biliary tree was reported in seven patients and detected a diaphragmatic defect in 85.71% of cases (*n* = 6) (Table 4).

Eighteen patients had an infected bilothorax, and the most common organism isolated was *Escherichia coli* (*n* = 5), followed by *Klebsiella* (*n* = 4) (Table 4).

Only two of the 115 patients had a left-sided bilothorax.

A chest tube was placed in 79 out of 107 patients (73.83%, 95% CI 64.59-81.35). In addition to chest tube thoracostomy or thoracentesis, 53 surgical interventions were reported in 52 patients with ERCP and/or biliary drain being the most common procedure, which was reported at 47.16% (*n* = 25). Similarly, VATS was reported in 24.52% of cases (*n* = 13), fistula repair in 22.64% of cases (*n* = 12), and open thoracotomy in one patient (Table 4).

Ninety-seven patients (84.35%, 95% CI 76.42-89.95) recovered with treatment (Table 4).

## 4. Discussion

In this review, we found 123 studies that reported bilothorax, of which 115 met the criteria for quantitative analysis. The most common etiology was PTBD, followed by injury-related, and the prognosis was overall favorable with the institution of pleural fluid drainage. Chest CT was the most commonly used radiological investigation, and chest tube thoracostomy was the prevalent treatment modality.

Bilothorax seems to be underdiagnosed, requiring a high index of suspicion for an adequate diagnosis. A careful history with particular attention to any surgical manipulation, radiation, or infection of hepatobiliary structures can be the first clue to the diagnosis. The latency from the initial insult to the development of bilothorax varies from days to years [41, 93, 99]. In our case, the bilothorax occurred about a month after laparoscopic cholecystectomy. Fortunately, the mortality from bilothorax remains low. In our review, around 84% of cases had favorable outcomes. Those with associated mortality had hepatobiliary or gastric carcinoma and succumbed to complications other than the bilious pleural effusion per se. The mainstay of the treatment was the drainage of the bilothorax, mostly through a chest tube. Surgical interventions were mentioned in 52 (45%) patients, and the most common procedure needed was biliary decompression. Surgical intervention was indicated if the bilothorax failed to resolve after placing the chest tube.

A bilopleural fistula was reported in 51% of the patients. It was diagnosed either with radiological investigation, during the VATS procedure, ERCP, or through dye injected in the pleural or abdominal cavity. Only 12 patients needed repair of the fistula which accounted for only 10% of the patients. The most common radiological investigation used was a CT scan, which detected BPF in only 25% of the cases. Of those 14 positive CT scans, three patients had biliary stents that transverse through the diaphragm resulting in bilothorax. HIDA scan, or MR study of hepatopancreatobiliary structure, was more sensitive in detecting a BPF. Our patient was investigated with a chest CT scan, which showed no diaphragmatic defect. The mechanism of bilothorax is poorly understood. A bilopleural fistula was present in 51% of the patients, but in the remaining 49% of the patients, no diaphragmatic defects were present. It is possible that bile might have been sucked into the pleural cavity through congenital microdefects in the diaphragm during negative intrathoracic pressure, similar to that of hepatic hydrothorax. These defects are usually not detected with a CT scan or during the VATS procedure.

The mean pleural fluid LDH level was 2650 IU/L, and TP was 4.25 g/dL, consistent with an exudative process. Some studies suggested that the pleural fluid to serum fluid bilirubin ratio could be used to differentiate exudative pleural effusion from transudative effusion, especially in resource-limited settings. The cutoff ratio suggested was less than 1 [124, 125]. The presence of bilirubin in the pleural space causes a cascade of inflammatory responses. This can lead to potential loculated pleural effusions [3] and also respiratory compromises like hypoxic respiratory failure or acute respiratory distress syndrome (ARDS) [62]. In our study, the mean PB/SB was 3.47. The mean WBC count was 4540/mm^3^, with mostly neutrophils predominant. Only 18 cases reported organisms grown from the pleural fluid, which suggests that leukocytosis is likely a result of inflammation induced by bile in the pleural fluid and not necessarily related to infection.

Our study had some limitations. The quality of the evidence was low, as the identified studies were case reports and case series. Most of the case reports lacked additional pleural fluid studies and information on cultures and cytology. Despite the low level of quality of evidence, this is the first systematic literature review involving four databases and is expected to help clinicians diagnose and treat bilothorax. Secondly, we included the abstracts presented at reputed societal conferences, which is again low-quality evidence, but this was done to minimize publication bias. Another limitation was the lack of SARS-CoV-2 PCR testing in the pleural fluid of the patient we reported. However, pleural effusion due to SARS-CoV-2 infection is relatively rare, with an incidence of around 2-11%, and is mostly bilateral. It is a late complication that appears around three to four weeks, is seen with severe parenchymal involvement, and carries a worse outcome [126]. Our patient had unilateral pleural effusion and did not have the parenchymal involvement that is commonly seen with COVID-19 pneumonia. We strongly believe this pleural effusion was unrelated to her concurrent SARS-CoV-2 infection.

There are no guidelines or consensus on how to treat bilothoraces, and based on the results of our review, we suggest that chest tube drainage should be the first line of treatment, with testing for the presence of infection with pleural fluid culture. If this is inadequate, nuclear studies should be done to investigate the presence of diaphragmatic defects. One should not rely on a CT scan of the chest or abdomen for the diagnosis of the BPF, as the yield seemed low. Then, surgical consultation to correct the existing BPF should be obtained for persistent bilious pleural effusion or large diaphragmatic defects seen on the radiological scans.

## 5. Conclusion

Bilothorax should be considered in new-onset pleural effusions, particularly of the right side, in patients with a history of surgery, trauma, radiation, or infection of the hepatobiliary structure. The measurement of pleural fluid and serum bilirubin level usually confirms the diagnosis. Treatment is generally done with drainage of bilious pleural effusion, preferably with a chest tube. The presence of a bilopleural fistula plays a role in determining the need for surgical correction.

## Figures and Tables

**Table 1:** Descriptive analysis of abstracts and case reports.

Authors	Year	Age	Sex	Background	Pleural fluid bilirubin	Serum bilirubin	Bilopleural fistulation	Pleural fluid studies	Organisms isolated	Imaging	Management	Outcome
Abstracts												
Aneja et al. [2]	2020	52	M	Laparoscopic sleeve gastrectomy	2.6	0.9	No	LDH: 960		CT: pleural effusion	Chest tube	Recovery
Austin et al. [3]	2016	59	M	Pancreatic adenocarcinoma with PTBD	8.8	7.5	No	LDH: 1170 Complex effusion		CT: loculated effusion HIDA: negative	Tube thoracostomy	Recovery
Bilal et al. [4]	2015	71	M	PTBD	n/a	n/a	No	1 L bilious pleural effusion drained		CT: biliary drain in the pleural space	Thoracentesis and antibiotics	Death
Celis et al. [5]	2015	45	F	PTBD	5	2	Yes	LDH: 1850		CT: pleural effusion	Chest tube, thoracostomy decortication, and antibiotics	Recovery
Coombs et al. [6]	2022	F	75	Abdominal surgery complicated by intra-abdominal bile leak	2.6	0.5	No		*Enterobacter cloacae* *Enterococcus faecalis*	CT: pleural effusion	Pigtail chest tube and IV antibiotics	Recovery
Fakih et al. [7]	2017	52	M	Gangrenous cholecystitis and lap chole	0.6	0.3	No	Lymphocytic predominant effusion		CT Abd: intrahepatic abscess	Chest tube	Recovery
Harcha et al. [8]	2022	63	F	Pancreatic head mass	n/a	11	No			CT: pleural effusion	None	Death (DNR)
Hayat and Oweis [9]	2021	69	M	Acute cholecystitis with laparoscopic cholecystectomy	6.4	4.8	No			CT: pleural effusion MRCP: fistula in the diaphragm	Chest tube and ERCP and COB stent	Recovery
Hossain and Lee [10]	2020	39	F	Percutaneous liver biopsy	n/a	n/a	No	*PB*/*SB* > 1		CT: pleural effusion	Thoracentesis	Recovery
Husari and El Kara [11]	2016	26	F	Liposuction of abdominal fat	16.3	n/a	Yes			CT and MRI: 2 tracts in the diaphragm	Chest tube and ex lap	Recovery
Kaya et al. [12]	2006	35	M	Hemigastrectomy with gastrojejunostomy	n/a	n/a	No			CT: pleural effusion	Serial thoracentesis	Recovery
Khan et al. [13]	2018	62	F	PTBD and CBD stent placement	16	0.9	No		*Klebsiella* *Streptococcus*	CT: pleural effusion	VATS and long decortication	Recovery
Maniak and Hawkins [14]	2020	51	F	PTBD	7.4	7.5	No	WBC: 635 LDH: 239 TP: 2.6		CT: pleural effusion	Thoracentesis and biliary drain removal	Recovery
Motika [15]	2008	66	M	Hepatocellular carcinoma	18	1.7	Yes	WBC: 2689, N96% LDH: 1195		MRCP: erosion of liver capsule	Chest tube and pleural decortication	Recovery
Núñez et al. [16]	2021	60	M	Left chest trauma with ribs fracture	4.2	0.17	No	Exudative mononuclear			Chest tube	Recovery
Olmstead et al. [17]	2020	73	M	Cholecystitis and open cholecystectomy	n/a	n/a	Yes	*PB*/*SB* > 1 Exudative			Chest tube and antibiotics	Recovery
Patel and Ly [18]	2016	66	M	Cholecystectomy	3.1	n/a	No				Bile duct stenting	Recovery
Patel et al. [19]	2018	40	F	Liver biopsy	2.6	n/a	No	*PB*/*SB* > 1			Chest tube	Recovery
Pew and Thomas [20]	2019	62	M	Orthotropic liver transplant	n/a	n/a	Yes	*PB*/*SB* > 1			Chest tube and fistula repair	Recovery
Poudel et al. [21]	2022	62	M	Laparoscopic cholecystectomy and right diaphragmatic hernia	21	1.6	No	WBC: 1500, L		HIDA: radioactivity in pleural space	Thoracentesis and ERCP stenting to biliary stump	Recovery
Rabold et al. [22]	2020	88	M	Laparoscopic cholecystectomy	1.7	0.9	No			CT: abdominal collection HIDA: normal	Chest tube and ERCP stenting	Recovery
Sun and Pagliarello [23]	2016	18	M	Penetrating injury to right hemithorax	n/a	n/a	Yes			HIDA: radioactivity in pleural space	Chest tube	Recovery
Talley [24]	2022	59	M	Gunshot injury to the abdomen	28.6	0.5	Yes			CT: suggestive of diaphragmatic track HIDA: radioactivity in pleural space	Chest tube and ERCP with biliary stent	Recovery
Won-seok and Jong-yul [25]	2018	33	M	PTBD	28.6	6.7	No	WBC: 13090, M93% LDH: 14041			Chest tube, ERCP with biliary stent, and VATS decortication	Recovery
Zhao et al. [26]	2019	62	M	PTBD and cholangitis	2.1	0.7	No				Chest tube	Recovery
Cases												
Addas et al. [27]	2021	50	M	PTBD for pancreatic carcinoma	n/a	n/a	Yes	*PB*/*SB* > 1	n/a	CT: biliary drain mispositioned to the pleural cavity	Chest tube	Death
Al-Qahtani [28]	2011	35	M	PTBD	16.78	Normal	No			CT: pleural effusion	Chest tube, antibiotics, and VATS	Recovery
Alvarenga et al. [29]	2019	25	M	Gunshot injury to the chest	25.7	n/a	Yes				Chest tube and surgery	Recovery
Armstrong and Taylor [30]	1982	64	F	PTBD	n/a	n/a	Yes	1 L green pleural fluid	*Escherichia coli*		None	Death (bilothorax diagnosed in autopsy)
*Austin et al.* [1]	*2017*	*59*	*M*	*Adenocarcinoma with PTBD*	*8.8*	*7.5*	*No*	*WBC: 190, N90%* *LDH: 1170*			*Chest tube*	*Death*
*Austin et al.* [1]	*2017*	*46*	*M*	*Cholangiocarcinoma with PTBD*	*7*	*7.9*	*No*	*WBC: 1125, N79%* *LDH: 131* *TP: 4.1*		*CT: biloma*	*Thoracentesis and perihepatic drain*	*Recovery*
*Austin et al.* [1]	*2017*	*53*	*M*	*Orthotropic liver transplant*	*4.9*	*4.8*	*Yes*	*N90%* *LDH: 239* *TP:3.6*			*Thoracentesis and biliary drain*	*Recovery*
*Austin et al.* [1]	*2017*	*59*	*M*	*Gastric cancer and PTBD*	*1.7*	*1.3*	*No*	*WBC: 5899, N95%* *LDH: 474* *TP: 3.3*		*CT: stent through the diaphragm*	*Chest tube and biliary drain*	*Recovery*
*Austin et al.* [1]	*2017*	*79*	*M*	*Duodenal cancer and PTBD*	*3.2*	*2.3*	*No*	*LDH: 833* *TP: 2.4*			*Chest tube*	*Death*
Aydogan et al. [31]	2013	41	M	Laparoscopic cholecystectomy and intra-abdominal bile leak	9.1	Normal	No			CT: pleural effusion	Thoracentesis	Recovery
Ball et al. [32]	2009	27	M	Gunshot thoracoabdominal injury	n/a	n/a	Yes	1 L green pleural fluid drained		CT: thoracoabdominal abscess	Chest tube, antibiotics, and surgery	Recovery
Bamberger et al. [33]	1997	28	M	Gunshot injury to the thorax	72.1	5	Yes			HIDA: positive	Chest tube	Recovery
Basu et al. [34]	2010	45	F	Cholecystitis and laparoscopic cholecystectomy	n/a	No	No	2.5 L bilious effusion			Chest tube	Death
Begum et al. [35]	2015	28	M	Surgical correction of lacerated liver	6.8	Normal	Yes	Exudate WBC: 4300, neutrophilic			Serial thoracentesis	Recovery
Bhattacharya et al. [36]	2006	24	M	Stab injury to the right upper quadrant of the abdomen	n/a	n/a	Yes	2 L of bilious pleural effusion drained			Chest tube	Recovery
Bini et al. [37]	2004	73	M	Subtotal gastric resection for gastric adenocarcinoma	1.68	n/a	Yes		*Stenotrophomonas* (*Xanthomonas*) *maltophilia*		Chest tube and repair of fistula	Recovery
Brazinsky and Colt [38]	1993	75	M	Laparoscopic cholecystectomy with abdominal bile leak	1.1	1.2	No	WBC: 5800 LDH: 614 TP: 5.9	*Klebsiella pneumoniae*	CT: pleural effusion MRI: negative	Thoracoscopy and pleural adhesiolysis	Recovery
Brunaud et al. [39]	2000	49	M	Motor vehicle accident	n/a	4	Yes			CT: pleural effusion	Chest tube and ERCP	Recovery
Chand et al. [40]	2012	52	F	Cholangitis and *Clostridium perfringens* septicemia	7.1	n/a	No	LDH: 2667		CT: 6 cm gas cavity above the right lobe of the liver	IV antibiotics	Death
Christensen et al. [41]	1974	22	M	Remote blunt trauma to right chest, sickle cell disease	4.5	7	No				Thoracentesis and IV antibiotics	Recovery
Cooper et al. [42]	2011	21	F	Trauma to right hemithorax	4.9	0.8	No			CT: pleural effusion	Chest tube	Recovery
Cosgun et al. [43]	2013	67	F	Cholecystectomy	0.26	0.17	Yes	WBC: 110, L LDH: 86 TP: 4.8 Exudate		MRC: contrast in the pleural cavity	Thoracentesis and ERCP papillotomy	Recovery
Dadlani et al. [44]	2021	71	M	Microwave ablation of hepatocellular carcinoma	n/a	n/a	Yes	*PB*/*SB* > 1		CT: defect in the diaphragm	Chest tube and repair of fistula	Recovery
Dahiya et al. [45]	2015	22	M	Gunshot injury to right chest	n/a	n/a	Yes			CT: pleural effusion HIDA: positive	Chest tube	Recovery
Dalvi et al. [46]	2006	85	M	ERCP and sphincterotomy	n/a	n/a	Yes			CT: pleural effusion	CBD stent	Recovery
Dasmahapatra and Pepper [47]	1988	56	F	Bile duct stenting migration	3.04	1.29	Yes			CT: stent through the diaphragm	Chest tube, decortication, and fistula repair	Recovery
Delande et al. [48]	2007	64	F	Cholangiocarcinoma s/p Whipple surgery	Biliary fluid	23.2	Yes		*Stenotrophomonas maltophila*		Chest tube and antibiotics	Death
Delcò et al. [49]	1994	75	M	Cholecystitis	27.94	0.53	Yes			CT: pleural effusion	Chest tube and repair of fistula	Recovery
Desai et al. [50]	2019	43	M	Right hepatectomy with a Roux-en-Y hepaticojejunostomy	n/a	n/a	Yes			CT: contrast through pigtail leaked into abdomen	Tube thoracostomy and decortication	Recovery
Dong-won et al. [51]	2012	88	M	PTBD and cholecystitis	1.52	0.79	Yes	WBC: 5280, N86% LDH: 124 TP: 2.6	*Escherichia coli*	CT: pleurobiliary fistula	Chest tube	Recovery
Dosik [52]	1975	29	M	Percutaneous liver biopsy	n/a	1.9	No				Chest tube	Recovery
Ellingsen et al. [53]	2016	50	F	Metastatic serous adenocarcinoma	3.1	n/a	No	WBC: 1128, N89% LDH: 1433 TP: 9.0		HIDA, MRI abdomen, and ERCP: normal	Thoracentesis and VATS	Recovery
Ezzeddine et al. [54]	2018	26	F	Liposuction including abdomen	16.3	1.9	Yes			CT: injury to liver, GB, and liver	Thoracentesis and repair of fistula	Recovery
Fayed and Hassan [55]	2018	58	F	Metastatic cholangiocarcinoma with PTBD	n/a	13.5	No	WBC: 4500, N95% LDH: 350 TP: 3.0		CT: pleural effusion	Chest tube	Recovery
Frampton et al. [56]	2010	64	M	Cholecystitis with CBD stone	10.53	n/a	Yes			MRCP: pleurobiliary fistula	Chest tube and CBD sphincterotomy	Recovery
Franklin and Mathai [57]	1980	13	F	Trauma to chest	n/a	1.3	Yes		No	No	Chest tube and surgical correction of diaphragmatic defect	Recovery
García Ruiz de Gordejuela et al. [58]	2007	52	M	Hepatopleural echinococcosis	n/a	n/a	Yes			CT: pleural effusion HIDA: positive	Chest tube and resection of the cyst	Recovery
Ghritlahaney [59]	2008	12	M	Trauma to chest	n/a	n/a	Yes		Coagulase-negative *Staphylococcus*	CT: tear in R diaphragm	Chest tube	Recovery
Gómez-Álvarez et al. [60]	2022	72	M	Ruptured cholecystitis	4.2	0.7	Yes	WBC: 470, N67% Exudate		CT: cholecystopleural fistula	Chest tube and surgical correction of the fistula	Recovery
Gorospe Sarasúa et al. [61]	2016	92	n/a	Recurrent cholecystitis with laparoscopic cholecystectomy	n/a	n/a	Yes			CT: abscess communicating with pleural space	Thoracentesis	Recovery
Hamers et al. [62]	2013	58	F	Liver lobectomy and radio ablation	20.7	n/a	No		*Enterococcus*	CT: effusion in subcapsular liver compartment	Chest tube and ERCP	Recovery
Herschman et al. [63]	1991	63	F	Choledocho-enterostomy for bile duct carcinoma	n/a	3.1	No				Chest tube	Death
Hsu et al. [64]	2001	66	M	CBD stone with pneumobilia	3.8	0.7	Yes	Exudate	*Escherichia coli* *Bacteroides* *Candida*	ERCP: fistula in the diaphragm	Chest tube with fibrinolytics	Recovery
Jain et al. [65]	2008	55	F	Gall bladder stones	n/a	n/a	Yes		*E. coli*	MRCP: fistulous tracks in the diaphragm	Chest tube with ERCP and ex lap	Recovery
Jenkinson et al. [66]	2013	60	F	Para-aortic lymph node sampling	12.6	2.2	No			CT: pleural effusion	Thoracentesis	Recovery
Jimeno Griñó et al. [67]	2019	90	M	Laparoscopic cholecystectomy	7.2	n/a	Yes	*PB*/*SB* > 1			Chest tube	Recovery
Karavdić et al. [68]	2018	10	F	Ultrasound-guided liver biopsy	14.5	Normal	No			CT: pleural effusion	Chest tube	Recovery
Karnik and Shair [69]	2019	62	M	Orthotopic liver transplant	18.5	Normal	No	Normal cell count LDH: 787			Chest tube and biliary stent	Recovery
Kerawala and Jamal [70]	2020	60	M	Metastasectomy of liver lesions	4.2	n/a	No	WBC: 17000			Chest tube, ERCP, and sphincterotomy	Recovery
Kim and Zangan [71]	2015	61	M	Orthotopic liver transplant	n/a	n/a	No				Thoracentesis	Death
Koide et al. [72]	2012	54	M	Chronic pancreatitis	7.3	0.91	No	LDH: 784 TP: 4.4 Hematocrit: 0.1% Exudative		CT: left pleural effusion	Thoracentesis	Recovery
Lee et al. [73]	2015	88	M	PTBD	1.52	0.59	No			CT: pleurobiliary track	Chest tube	Recovery
Lee et al. [74]	2007	79	M	PTBD under US guidance	16.7	1.1	Yes	LDH: 670	*Klebsiella pneumoniae* *E. coli*		Chest tube	Recovery
Lewis et al. [75]	2009	66	M	Transarterial chemoembolization for hepatocellular carcinoma	18.4	n/a	Yes				Chest tube and open thoracotomy with decortication	Recovery
Liberale et al. [76]	2004	65	F	Radio ablation of liver cancer	n/a	n/a	Yes			CT: duodenohepatopleural fistula	Chest tube and ERCP	Recovery
López-Garnica et al. [77]	2013	44	F	TIPS procedure followed by the liver transplant	7.4	3.1	Yes	LDH: 194		CT: pleural effusion HIDA: positive	Thoracentesis and ERCP	Recovery
Meristoudis et al. [78]	2021	51	M	Orthotopic liver transplant	16.5	0.5	Yes				Chest tube	Recovery
Mohammed et al. [79]	2017	79	F	Cholangiocarcinoma and portal vein embolization	n/a	n/a	Yes	WBC: high	Gram-negative rods		Chest tube and biliary drain	Death
Navsaria et al. [80]	2002	38	M	Gunshot injury to right hemithorax	n/a	n/a	Yes	WBC: 18000 Bilious effusion		CT: diaphragmatic defect	Chest tube and ERCP with sphincterotomy	Recovery
Newberg et al. [81]	1969	50	M	Hydatid cyst of liver	n/a	n/a	Yes				Chest tube and fistula repair	Recovery
Park et al. [82]	2008	64	F	Cholecystitis, PTBD, and biliary dilation	21.4	1.2	Yes			HIDA: radioactivity in pleural space	Chest tube and antibiotics	Recovery
Petri et al. [83]	2019	63	M	PTBD	4.9	3.7	Yes	WBC: 131, N97% LDH: 774			Chest tube and VATS	Death
Pisani and Zeller [84]	1990	59	F	Liver biopsy	76.1	29.3	No	WBC: 11, N60% LDH: 113			Serial thoracentesis	Death
Reddy et al. [85]	2019	43	F	Sickle cell crisis	3	2.2	No	WBC: 676 LDH: 160 TP: 8.2		CXR: left pleural effusion	Thoracentesis	Recovery
Robin et al. [86]	1990	36	M	Motor vehicle accident and congenital diaphragmatic hernia	n/a	n/a	Yes				Thoracentesis and repair of the defect	Recovery
Row [87]	1989	63	M	Partial gastrectomy	n/a	n/a	No				Chest tube and resection of ischemic bowel	Recovery
Seeman et al. [88]	2020	20	M	Gunshot injury	n/a	n/a	Yes				Chest tube and VATS decortication	Recovery
Seong et al. [89]	2010	76	M	Cholecystitis	11.7	0.33	Yes		*Staphylococcus hominis*		Chest tube	Recovery
Shah et al. [90]	2019	71	M	PTBD and cholecystitis	9.1	1.2	No	WBC: 771 LDH: 2810 TP: 2.9			Chest tube	Recovery
Sheik-Gafoor et al. [91]	1998	55	M	Gunshot injury to epigastrium	n/a	n/a	Yes			Nuclear scan: radioactivity in the pleural cavity	Chest tube	Recovery
*Sokouti et al.* [92]	*2010*	*67*	*M*	*Hydatid cyst of the liver*	*n/a*	*n/a*	*Yes*	*WBC: 11500, E5%* *Bilious effusion*		*CXR: calcified cyst in right liver lobe*	*Chest tube, open thoracotomy, decortication, and fistula correction*	Recovery
Soler-Sempere et al. [93]	2015	83	M	Left side cholecystectomy 8 years ago	5.8	0.4	No	WBC: 130, N65% LDH: 2551 TP: 5.8			ERCP sphincterotomy and biliary stent placement	Death
Srivali and De Giacomi [94]	2021	71	M	Cholecystitis	9.5	4.7	No				Chest tube	Recovery
*Strange et al.* [95]	*1988*	*59*	*M*	*PTBD*	*41.7*	*3.1*	*No*	WBC: 1800, N98% LDH: 1800 TP: 1.8			*Repositioning of PTBD and thoracentesis*	Recovery
*Strange et al.* [95]	*1988*	*76*	*F*	*PTBD*	*2.1*	*1.5*	*No*	WBC: 9280, N82% LDH: 332 TP: 3.5			*Thoracentesis and drain removal*	Recovery
Tesfaye et al. [96]	2022	30	M	Gunshot injury to the chest	n/a	n/a	Yes				Chest tube and fistula repair	Recovery
Truong ang Huaringa [97]	2013	79	F	Obstructive jaundice and PTBD	22.9	n/a	No		*Klebsiella pneumoniae*		Chest tube, VATS decortication, and antibiotics	Recovery
Turkington et al. [98]	2007	51	M	PTBD for advanced gastric adenocarcinoma	44.79	22.6	No				Chest tube	Recovery
van Niekerk et al. [99]	2017	76	M	Biliary sphincterotomy for gall bladder carcinoma	33.9	3.1	No			HIDA: radioactivity in pleural space	Thoracentesis	Death
Vrachliotis et al. [100]	2022	80	M	PTBD and periampullary cancer	n/a	15	No			CT: stents in pleural space	Chest tube and biliary stent	Recovery
Waelbers et al. [101]	2005	4	F	Traumatic chest injury	7.6	1	No			HIDA: positive	Chest tube and antibiotics	Recovery
Williams et al. [102]	1971	23	M	Trauma to the right abdomen	n/a	n/a	Yes				Chest tube, T-tube choledochostomy, and decompression of bile duct	Recovery
Wong et al. [103]	2019	41	M	Microwave ablation of the liver	3.4	0.5	No		*Actinomyces odontolyticus*		Chest tube and biliary drain	Recovery
*Wu et al.* [104]	*2020*	*62*	*M*	*Microwave ablation of liver cancer*	*n/a*	*n/a*	*Yes*	*WBC: 7500, N93%* *LDH: 10186*	*Escherichia coli*	*CT: diaphragmatic defect*	*Chest tube and antibiotics*	Recovery
*Wu et al.* [104]	*2002*	*46*	*M*				*Yes*	*LDH: 24783*		*CT: pleural effusion* *HIDA: positive*	*Chest tube and antibiotics*	Recovery
Yamazaki et al. [105]	2005	41	M	Pancreatic head and body pseudocysts	5.6	n/a	Yes				Chest tube, resection of pancreatic cysts, and pancreatojejunostomy	Recovery
Yankova and Hadjidekov [106]	2017	42	F	Liver transplant	n/a	13.98	Yes				Thoracentesis	Recovery
Yi-Yung et al. [107]	2018	53	F	Neuroendocrine tumor of the pancreatic head and PTBD	23.7	n/a	Yes		*Enterococcus* *Candida*		Chest tube and repair of BF	Death
Peng et al. [108]	2022	53	F	Left lobe liver resection and splenectomy a year ago	4	18	Yes			CT: pleural effusion HIDA: negative Fluorescence imaging: positive	Thoracentesis and conservative management	Death
Yokoe and Yamaguchi [109]	2019	73	F	Lung adenocarcinoma	7.6	2.9	No				Thoracentesis	Death

PTBD = percutaneous transhepatic biliary drainage; CBD = common bile duct; WBC = white cell count (per mm^3^); LDH = lactate dehydrogenase (IU/L); TP = total protein (g/dL); L = lymphocyte; N = neutrophil; CT = computerized tomographic scan (of the chest); MRI = magnetic resonance imaging; MRCP = magnetic resonance cholangiopancreatography; HIDA = hepatobiliary iminodiacetic acid; ERCP = endoscopic retrograde cholangiopancreatography; n/a = not available; PB/SB = pleural bilirubin/serum bilirubin. Italics are the patients pooled from case series and comprehensive reviews.

**Table 2:** Descriptive analysis of observational studies.

Authors	Year	Study	Patients	Background	Pleural fluid studies	Bilopleural fistulation
Amir-Jahed et al. [110]	1972	Retrospective cohort	10	Hepatic echinococcosis and amebiasis	n/a	Yes
Andrade-Alegre and Ruiz-Valdes[111]	2013	Retrospective cohort	5	Traumatic chest injury	*Bilirubin* = 16.24 (average)	Yes
Austin et al. [1]	2015	Case series	5	Upper GI malignancies	*PB*/*SB* ≥ 1	n/a
Clark et al. [112]	1981	Retrospective cohort	1 of 42	PTBD	n/a	n/a
Carter [113]	1987	Retrospective	1 of 51	Traumatic chest injury	n/a	n/a
Ciriaco et al. [114]	2006	Case series	3	Traumatic chest injury	n/a	Yes
Demers et al. [115]	2013	Case series	1 of 4	Percutaneous thermal ablation of liver cancer	n/a	Yes
Feld et al. [116]	1997	Case series	2 of 3	Gunshot injury to the thorax	*Bilirubin* = 24.9 (only 1 patient reported)	Yes
Gil et al. [117]	2008	Retrospective	1 of 38	Balloon dilation of papilla for clearance of CBD stone	n/a (biliary pleural effusion)	No
Ivatury et al. [118]	1984	Case series	3	Traumatic chest injury	n/a	Yes
Najjar et al. [119]	2018	Retrospective	36	n/a	n/a	n/a
Sano and Yotsumoto [120]	2016	Case series	2	PTBD	*Bilirubin* = 57.78, other n/a	n/a
Sastre et al. [121]	2021	Retrospective study	7	Penetrating trauma of the thoracic and abdominal wall	Presence of bilirubin in the pleural fluid	Yes
Singh et al. [122]	2002	Retrospective study	3 of 8	Abdominal trauma and percutaneous transhepatic cholangiography	Bilious effusion	Yes
Sood et al. [123]	2021	Retrospective study	1 of 10	Bile leak following gunshot injury	Bilious pleural effusion	n/a
Sokouti et al. [92]	2010	Case series	1 of 2	Hydatid cyst of the liver	Bilious effusion	Yes
Strange et al. [95]	1998	Case series	2	Percutaneous biliary drainage	Bilious effusion	No
Wu et al. [104]	2020	Case series	2	Microwave ablation of liver cancer	Bilious effusion	Yes

GI = gastrointestinal; PTBD = percutaneous transhepatic biliary drainage; PB/SB = pleural bilirubin/serum bilirubin; n/a = not available.

**Table 3:** Quantitative analysis of the pooled patients—part I.

	Observation (*n*)	Mean	SD	Frequency	Percentage		95% CI
Age	115	54.26	19.60				
Pleural fluid B	72	72	12.19				
Serum B	61	61	4.44				
Ratio						3.47	2.15-4.7
WBC (k/*μ*L)	25	4540.2	5383.56				
LDH (IU/L)	27	2650	5412.86				
TP (g/dL)	16	4.25	2.05				
Sex	114			77 (male)	67.54		
Background	115			97 (surgical)	84.35		76.42-89.95
BPF	115			59	51.30		42.13-60.39
Organisms	18			18	15.65		
Imaging	68			36	52.94		40.92-64.62
Management	107			79 (chest tube)	73.83		64.59-81.35
Outcome	115			97 (recovery)	84.34		76.42-89.95
Laterality	115			113 (right side)	92.26		
Surgical management	52			52	45.27		
Case reports	123			80	65.04		
Abstracts	123			25	20.32		
Studies	123			18	14.63		

SD = standard deviation; CI = confidence interval; WBC = white cell count; LDH = lactate dehydrogenase; TP = total protein; BPF = bilopleural fistula.

**Table 4:** Quantitative analysis of the pooled patients—part II.

	Total number reported (*n*)		Frequency	Percentage	
Background	115	PTBD	27	23.47	
		Gunshot or trauma	18	15.65	
		Liver transplant	6	5.21	
		Hydatid cyst	3	2.60	
		Liposuction	2	1.73	

Surgery	53	VATS	13	24.52	
		Biliary drain/ERCP/biliary stents	25	47.16	
		Fistula repair	12	22.64	
		Open thoracotomy	1	1.88	
		Unspecified	1	1.88	

Organisms	18	*Escherichia coli*	5	27.77	
		*Klebsiella*	4	22.22	
		*Enterococcus*	3	16.66	
		*Candida*	3	11.11	

Imaging	73			Positive frequency	Percentage
		CT scan	51	14	25.92
		HIDA scan	14	11	78.57
		MR study	7	6	85.71
		Other nuclear studies	1	1	100

PTBD = percutaneous transhepatic biliary drainage; VATS = video-assisted thoracic surgery; ERCP = endoscopic retrograde cholangiopancreatography; HIDA = hepatobiliary iminodiacetic acid; MR = magnetic resonance.

## Supplementary Materials

Supplementary 1 PRISMA checklist.

Supplementary 2 Search strategy.

Supplementary 3 Risk of bias assessment.
