# Supplementary material for: Bilothorax: A Case Report and Systematic Literature Review of the Rare Entity
Source: Pulm Med. 2024 Jun 21;2024:3973056. doi: 10.1155/2024/3973056 (PMC11213635; doi:10.1155/2024/3973056)
Supplement: Supplementary 2 — Search strategy. [file 3973056.f2.docx]

**Search strategy (Jan 19, 2023)**

| **Database** | **Boolean Parameters** | **Hits** | **Link** |
| --- | --- | --- | --- |
| Pubmed/Medline | “bilothorax” OR “cholethorax” OR “biliary pleural effusion” OR “bilious pleural effusion” OR "thoracobilia" | 329 | <https://pubmed.ncbi.nlm.nih.gov/?term=%E2%80%9Cbilothorax%E2%80%9D+OR+%E2%80%9Ccholethorax%E2%80%9D+OR+%E2%80%9Cbiliary+pleural+effusion%E2%80%9D+OR+%E2%80%9Cbilious+pleural+effusion%E2%80%9D+OR+%22thoracobilia%22&sort=date&size=200> |
| Google Scholar | “bilothorax” OR “cholethorax” OR “biliary pleural effusion” OR “bilious pleural effusion” OR "thoracobilia" | 410 | <https://scholar.google.com/scholar?hl=en&as_sdt=0%2C47&q=%E2%80%9Cbilothorax%E2%80%9D+OR+%E2%80%9Ccholethorax%E2%80%9D+OR+%E2%80%9Cbiliary+pleural+effusion%E2%80%9D+OR+%E2%80%9Cbilious+pleural+effusion%E2%80%9D+OR+%22thoracobilia%22&btnG=> |
| CINAHL | “bilothorax” OR “cholethorax” OR “biliary pleural effusion” OR “bilious pleural effusion” OR "thoracobilia" | 20 | <https://web.s.ebscohost.com/ehost/resultsadvanced?vid=2&sid=d42dad25-26d0-4c1a-bdd9-988b17e0e129%40redis&bquery=%e2%80%9cbilothorax%e2%80%9d+OR+%e2%80%9ccholethorax%e2%80%9d+OR+%e2%80%9cbiliary+pleural+effusion%e2%80%9d+OR+%e2%80%9cbilious+pleural+effusion%e2%80%9d+OR+%22thoracobilia%22&bdata=JmRiPWNjbSZhdXRodHlwZT1zc28mY3VzdGlkPXMxMjEwMjQ5JnR5cGU9MSZzZWFyY2hNb2RlPVN0YW5kYXJkJnNpdGU9ZWhvc3QtbGl2ZSZzY29wZT1zaXRl> |
| Embase | “bilothorax” OR “cholethorax” OR “biliary pleural effusion” OR “bilious pleural effusion” OR "thoracobilia" | 78 | <https://www.embase.com/#advancedSearch/resultspage/history.1/page.1/25.items/orderby.date/source>. |

Supplementary Table 2: Search strategy
