# Supplementary material for: Bilothorax: A Case Report and Systematic Literature Review of the Rare Entity
Source: Pulm Med. 2024 Jun 21;2024:3973056. doi: 10.1155/2024/3973056 (PMC11213635; doi:10.1155/2024/3973056)
Supplement: Supplementary 3 — Risk of bias assessment. [file 3973056.f3.docx]

| **Authors** | **Q1** | **Q2** | **Q3** | **Q4** | **Q5** | **Q6** | **Q7** | **Q8** | **Total** |
| --- | --- | --- | --- | --- | --- | --- | --- | --- | --- |
| Abstracts | | | | | | | | | |
| Aneja ^2^ | 1 | 1 | 1 | 1 | 1 | 1 | 0 | 0 | 6 |
| Austin ^3^ | 1 | 1 | 1 | 1 | 1 | 1 | 0 | 0 | 6 |
| Bilal ^4^ | 1 | 1 | 1 | 0 | 1 | 1 | 0 | 0 | 5 |
| Celis ^5^ | 1 | 1 | 1 | 1 | 1 | 1 | 1 | 0 | 7 |
| Coombs ^6^ | 1 | 1 | 1 | 1 | 1 | 1 | 1 | 0 | 7 |
| Faikh ^7^ | 1 | 1 | 1 | 0 | 1 | 1 | 1 | 0 | 5 |
| Haracha ^8^ | 1 | 1 | 1 | 0 | 0 | 0 | 1 | 0 | 4 |
| Hayat ^9^ | 1 | 1 | 1 | 1 | 1 | 1 | 1 | 0 | 7 |
| Hossain ^10^ | 1 | 1 | 1 | 0 | 1 | 1 | 1 | 0 | 6 |
| Husari ^11^ | 1 | 1 | 1 | 0 | 1 | 1 | 1 | 0 | 6 |
| Kaya ^12^ | 1 | 1 | 1 | 0 | 1 | 1 | 1 | 0 | 6 |
| Khan ^13^ | 1 | 1 | 1 | 0 | 1 | 1 | 1 | 0 | 6 |
| Maniak ^14^ | 1 | 1 | 1 | 1 | 1 | 1 | 1 | 0 | 7 |
| Motika ^15^ | 1 | 1 | 1 | 1 | 1 | 1 | 1 | 0 | 6 |
| Nunez ^16^ | 1 | 1 | 1 | 1 | 1 | 1 | 1 | 0 | 6 |
| Olmstead ^17^ | 1 | 1 | 1 | 0 | 1 | 1 | 1 | 0 | 6 |
| Patel J ^18^ | 1 | 1 | 1 | 0 | 1 | 1 | 1 | 0 | 7 |
| Patel K ^19^ | 1 | 1 | 1 | 0 | 1 | 1 | 1 | 0 | 6 |
| Pew ^20^ | 1 | 1 | 1 | 0 | 1 | 1 | 1 | 0 | 6 |
| Poudel ^21^ | 1 | 1 | 1 | 0 | 1 | 1 | 1 | 0 | 6 |
| Rabold ^22^ | 1 | 1 | 1 | 1 | 1 | 1 | 1 | 0 | 7 |
| Sun ^23^ | 1 | 1 | 1 | 0 | 1 | 1 | 1 | 0 | 6 |
| Talley ^24^ | 1 | 1 | 1 | 1 | 1 | 1 |  | 1 | 7 |
| Won-seok ^25^ | 1 | 1 | 1 | 1 | 1 | 1 | 1 | 0 | 6 |
| Zhao ^26^ | 1 | 1 | 1 | 1 | 1 | 1 | 1 | 0 | 6 |
| Case Reports | | | | | | | | | |
| Addas ^27^ | 1 | 1 | 1 | 0 | 1 | 1 | 1 | 0 | 6 |
| Al-Qahtani ^28^ | 1 | 1 | 1 | 0 | 1 | 1 | 1 | 0 | 6 |
| Alvarenga ^29^ | 1 | 1 | 1 | 0 | 1 | 1 | 1 | 0 | 6 |
| Armstrong ^30^ | 1 | 1 | 1 | 1 | 1 | 1 | 1 | 0 | 7 |
| *Austin* ^1^ | 1 | 1 | 1 | 1 | 1 | 1 | 1 | 0 | 7 |
| Aydogan ^31^ | 1 | 1 | 1 | 0 | 1 | 1 | 1 | 0 | 6 |
| Ball ^32^ | 1 | 1 | 1 | 0 | 1 | 1 | 1 | 0 | 6 |
| Bamberger ^33^ | 1 | 1 | 1 | 1 | 1 | 1 | 1 | 0 | 7 |
| Basu ^34^ | 1 | 1 | 1 | 0 | 1 | 1 | 1 | 0 | 6 |
| Begum ^35^ | 1 | 1 | 1 | 0 | 1 | 1 | 1 | 0 | 6 |
| Bhattacharya ^36^ | 1 | 1 | 1 | 0 | 1 | 1 | 1 | 0 | 6 |
| Bini ^37^ | 1 | 1 | 1 | 0 | 1 | 1 | 1 | 1 | 7 |
| Brazinsky ^38^ | 1 | 1 | 1 | 1 | 1 | 1 | 1 | 0 | 7 |
| Brunaud ^39^ | 1 | 1 | 1 | 0 | 1 | 1 | 1 | 0 | 6 |
| Chand ^40^ | 1 | 1 | 1 | 0 | 1 | 1 | 1 |  | 6 |
| Christensen ^41^ | 1 | 1 | 1 | 0 | 1 | 1 | 1 | 0 | 6 |
| Cooper ^42^ | 1 | 1 | 1 | 0 | 1 | 1 | 1 | 0 | 6 |
| Cosgun ^43^ | 1 | 1 | 1 | 1 | 1 | 1 | 1 | 0 | 6 |
| Dadlani ^44^ | 1 | 1 | 1 | 0 | 1 | 1 | 1 | 0 | 6 |
| Dahiya ^45^ | 1 | 1 | 1 | 0 | 1 | 1 | 1 | 0 | 6 |
| Dalvi ^46^ | 1 | 1 | 1 | 0 | 1 | 1 | 1 | 0 | 6 |
| Dasmahapatra ^47^ | 1 | 1 | 1 | 1 | 1 | 1 | 1 | 0 | 7 |
| Delande ^48^ | 1 | 1 | 1 | 0 | 1 | 1 | 1 | 0 | 6 |
| Delco ^49^ | 1 | 1 | 1 | 1 | 1 | 1 | 1 | 0 | 7 |
| Desai ^50^ | 1 | 1 | 1 | 0 | 1 | 1 | 1 | 0 | 6 |
| Dong ^51^ | 1 | 1 | 1 | 1 | 1 | 1 | 1 | 0 | 7 |
| Dosik ^52^ | 1 | 1 | 1 | 0 | 1 | 1 | 1 | 0 | 6 |
| Ellingsen ^53^ | 1 | 1 | 1 | 0 | 1 | 1 | 1 | 0 | 6 |
| Ezzeddine ^54^ | 1 | 1 | 1 | 1 | 1 | 1 | 1 | 0 | 7 |
| Fayed ^55^ | 1 | 1 | 1 | 0 | 1 | 1 | 1 | 0 | 6 |
| Frampton ^56^ | 1 | 1 | 1 | 0 | 1 | 1 | 1 | 0 | 6 |
| Franklin ^57^ | 1 | 1 | 1 | 0 | 1 | 1 | 1 | 0 | 6 |
| García ^58^ | 1 | 1 | 1 | 0 | 1 | 1 | 0 | 0 | 5 |
| Ghritlahaney ^59^ | 1 | 1 | 1 | 0 | 1 | 1 | 0 | 0 | 5 |
| Gomez ^60^ | 1 | 1 | 1 | 1 | 1 | 1 | 1 | 0 | 7 |
| Gorospe ^61^ | 1 | 1 | 1 | 0 | 1 | 1 | 0 | 0 | 5 |
| Hamers ^62^ | 1 | 1 | 1 | 0 | 1 | 1 | 0 | 0 | 5 |
| Herschman ^63^ | 1 | 1 | 1 | 1 | 1 | 1 | 0 | 0 | 6 |
| Hsu ^64^ | 1 | 1 | 1 | 1 | 1 | 1 | 1 | 0 | 7 |
| Jain ^65^ | 1 | 1 | 1 | 0 | 1 | 1 | 0 | 0 | 5 |
| Jenkinson ^66^ | 1 | 1 | 1 | 1 | 1 | 1 | 1 | 0 | 7 |
| Jimeno ^67^ | 1 | 1 | 1 | 0 | 1 | 1 | 0 | 0 | 5 |
| Karavdić ^68^ | 1 | 1 | 1 | 0 | 1 | 1 | 0 | 0 | 5 |
| Karnik ^69^ | 1 | 1 | 1 | 0 | 1 | 1 | 0 | 0 | 5 |
| Kerawala ^70^ | 1 | 1 | 1 | 0 | 1 | 1 | 1 | 0 | 6 |
| Kim ^71^ | 1 | 1 | 1 | 0 | 0 | 0 | 0 | 0 | 3 |
| Koide ^72^ | 1 | 1 | 1 | 1 | 1 | 1 | 1 | 0 | 7 |
| Lee ^73^ | 1 | 1 | 1 | 1 | 1 | 1 | 1 | 0 | 7 |
| Lee M-T ^74^ | 1 | 1 | 1 | 1 | 1 | 1 | 1 | 0 | 7 |
| Lewis ^75^ | 1 | 1 | 1 | 0 | 1 | 1 | 0 | 0 | 5 |
| Liberale ^76^ | 1 | 1 | 1 | 0 | 1 | 1 | 0 | 0 | 5 |
| López-Garnica ^77^ | 1 | 1 | 1 | 1 | 1 | 1 | 1 | 0 | 7 |
| Meristoudis ^78^ | 1 | 1 | 1 | 1 | 1 | 1 | 0 | 0 | 6 |
| Mohammed ^79^ | 1 | 1 | 1 | 0 | 1 | 1 | 0 | 0 | 5 |
| Navsaria ^80^ | 1 | 1 | 1 | 0 | 1 | 1 | 0 | 0 | 5 |
| Newberg ^81^ | 1 | 1 | 1 | 0 | 1 | 1 | 0 | 0 | 5 |
| Park ^82^ | 1 | 1 | 1 | 1 | 1 | 1 | 0 | 0 | 6 |
| Petri ^83^ | 1 | 1 | 1 | 1 | 1 | 1 | 1 | 0 | 7 |
| Pisani ^84^ | 1 | 1 | 1 | 1 | 1 | 1 | 1 | 0 | 7 |
| Reddy ^85^ | 1 | 1 | 1 | 1 | 1 | 1 | 1 | 0 | 7 |
| Robin ^86^ | 1 | 1 | 1 | 0 | 1 | 1 | 0 | 0 | 5 |
| Row ^87^ | 1 | 1 | 1 | 0 | 1 | 1 | 0 | 0 | 5 |
| Seeman ^88^ | 1 | 1 | 1 | 0 | 1 | 1 | 0 | 0 | 5 |
| Seong ^89^ | 1 | 1 | 1 | 1 | 1 | 1 | 1 | 0 | 7 |
| Shah ^90^ | 1 | 1 | 1 | 1 | 1 | 1 | 1 | 0 | 7 |
| Sheik-Gafoor ^91^ | 1 | 1 | 1 | 0 | 1 | 1 | 0 | 0 | 5 |
| *Sokouti* ^92^ | 1 | 1 | 1 | 0 | 1 | 1 | 0 | 0 | 5 |
| Soler ^93^ | 1 | 1 | 1 | 1 | 1 | 1 | 1 | 0 | 7 |
| Srivali ^94^ | 1 | 1 | 1 | 1 | 1 | 1 | 0 | 0 | 6 |
| *Strange* ^95^ | 1 | 1 | 1 | 1 | 1 | 1 | 1 | 0 | 7 |
| Tesfaye ^96^ | 1 | 1 | 1 | 0 | 1 | 1 | 0 | 0 | 5 |
| Truong ^97^ | 1 | 1 | 1 | 0 | 1 | 1 | 0 | 0 | 5 |
| Turkington ^98^ | 1 | 1 | 1 | 1 | 1 | 1 | 1 | 0 | 7 |
| Van Niekerk ^99^ | 1 | 1 | 1 | 1 | 1 | 1 | 1 | 0 | 6 |
| Vrachliotis ^100^ | 1 | 1 | 1 | 0 | 1 | 1 | 0 | 0 | 5 |
| Waelbers ^101^ | 1 | 1 | 1 | 0 | 1 | 1 | 0 | 0 | 5 |
| Williams ^102^ | 1 | 1 | 1 | 0 | 1 | 1 | 0 | 0 | 5 |
| Wong ^103^ | 1 | 1 | 1 | 1 | 1 | 1 | 1 | 0 | 7 |
| Yamazaki ^105^ | 1 | 1 | 1 | 0 | 1 | 1 | 0 | 0 | 5 |
| Yankova ^106^ | 1 | 1 | 1 | 0 | 1 | 1 | 0 | 0 | 5 |
| Yi-Yung ^107^ | 1 | 1 | 1 | 0 | 1 | 1 | 1 | 0 | 6 |
| Yisheng (Peng) ^108^ | 1 | 1 | 1 | 1 | 1 | 1 | 0 | 0 | 6 |
| Yokoe ^109^ | 1 | 1 | 1 | 1 | 1 | 1 | 1 | 0 | 7 |

Supplementary Table 3: The Joanna Briggs Institute’s critical appraisal for case reports

|  | Yes | No | Unclear | Not applicable |
| --- | --- | --- | --- | --- |
| 1. Were patient’s demographic characteristics clearly described? | □ | □ | □ | □ |
| 1. Was the patient’s history clearly described and presented as a timeline? | □ | □ | □ | □ |
| 1. Was the current clinical condition of the patient on presentation clearly described? | □ | □ | □ | □ |
| 1. Were diagnostic tests or assessment methods and the results clearly described? | □ | □ | □ | □ |
| 1. Was the intervention(s) or treatment procedure(s) clearly described? | □ | □ | □ | □ |
| 1. Was the post-intervention clinical condition clearly described? | □ | □ | □ | □ |
| 1. Were adverse events (harms) or unanticipated events identified and described? | □ | □ | □ | □ |
| 1. Does the case report provide takeaway lessons? | □ | □ | □ | □ |

The Joanna Briggs Institute’s critical appraisal checklist for case reports

<https://jbi.global/critical-appraisal-tools>

| **Authors** | **Q1** | **Q2** | **Q3** | **Q4** | **Q5** | **Q6** | **Q7** | **Q8** | **Q9** | **Q10** | **Total** |
| --- | --- | --- | --- | --- | --- | --- | --- | --- | --- | --- | --- |
| Austin ^1^ | 1 | 1 | 1 | 1 | 1 | 1 | 1 | 0 | 1 | 1 | 8 |
| Ciriaco ^114^ | 1 | 1 | 1 | 1 | 1 | 1 | 0 | 0 | 0 | 0 | 7 |
| Demers ^115^ | 1 | 0 | 1 | 1 | 1 | 1 | 1 | 0 | 0 | 0 | 6 |
| Feld ^116^ | 1 | 0 | 1 | 1 | 1 | 1 | 0 | 0 | 0 | 1 | 6 |
| Ivatury ^118^ | 1 | 0 | 1 | 1 | 0 | 1 | 0 | 0 | 0 | 1 | 5 |
| Sokouti ^92^ | 1 | 0 | 0 | 1 | 0 | 1 | 1 | 0 | 0 | 1 | 5 |
| Strange ^95^ | 1 | 1 | 1 | 1 | 0 | 1 | 1 | 0 | 0 | 1 | 7 |
| Wu ^104^ | 1 | 1 | 1 | 1 | 0 | 1 | 1 | 0 | 1 | 1 | 8 |

Supplementary Table 4: The Joanna Briggs Institute’s critical appraisal for case-series

|  | Yes | No | Unclear | Not applicable |
| --- | --- | --- | --- | --- |
| 1. Were there clear criteria for inclusion in the case series? | □ | □ | □ | □ |
| 2. Was the condition measured in a standard, reliable way for all participants included in the case series? | □ | □ | □ | □ |
| 3. Were valid methods used for identification of the condition for all participants included in the case series? | □ | □ | □ | □ |
| 4. Did the case series have consecutive inclusion of participants? | □ | □ | □ | □ |
| 5. Did the case series have complete inclusion of participants? | □ | □ | □ | □ |
| 6. Was there clear reporting of the demographics of the participants in the study? | □ | □ | □ | □ |
| 7. Was there clear reporting of clinical information of the participants? | □ | □ | □ | □ |
| 8. Were the outcomes or follow up results of cases clearly reported? | □ | □ | □ | □ |
| 9. Was there clear reporting of the presenting site(s)/clinic(s) demographic information? | □ | □ | □ | □ |
| 10. Was statistical analysis appropriate? | □ | □ | □ | □ |

The Joanna Briggs Institute’s critical appraisal checklist for case-series

<https://jbi.global/critical-appraisal-tools>

| **Authors** | **Q1** | **Q2** | **Q3** | **Q4** | **Q5** | **Q6** | **Q7** | **Q8** | **Q9** | **Q10** | **Q11** | **Total** |
| --- | --- | --- | --- | --- | --- | --- | --- | --- | --- | --- | --- | --- |
| Amir-Jahed ^110^ | 1 |  | 0 | 0 | 0 | 0 | 1 | 0 | 0 | 0 | 1 | 3 |
| Andrade-Alegre ^111^ | 0 | 0 | 1 | 0 | 0 | 0 | 0 | 0 | 0 | 0 | 1 | 2 |
| Clark ^112^ | 0 | 0 | 0 | 0 | 0 | 0 | 1 | 0 | 0 | 0 | 1 | 2 |
| Carter ^113^ | 0 | 0 | 0 | 0 | 0 | 0 | 1 | 0 | 0 | 0 | 1 | 2 |
| Gil ^117^ | 0 | 0 | 0 | 0 | 0 | 0 | 1 | 0 | 0 | 0 | 1 | 2 |
| Najjar ^119^ | 1 | 1 | 1 | 0 | 0 | 0 | 1 | 0 | 0 | 0 | 0 | 4 |
| Sastre ^121^ | 1 | 0 | 1 | 0 | 0 | 0 | 1 | 0 | 0 | 0 | 0 | 4 |
| Singh ^122^ | 0 | 0 | 1 | 0 | 0 | 0 | 1 | 0 | 0 | 0 | 0 | 2 |
| Sood ^123^ | 0 | 0 | 0 | 0 | 0 | 1 | 0 | 0 | 0 | 0 | 0 | 1 |

Supplementary Table 5: The Joanna Briggs Institute’s critical appraisal for cohort studies

|  | Yes | No | Unclear | Not applicable |
| --- | --- | --- | --- | --- |
| 1. Were the two groups similar and recruited from the same population? | □ | □ | □ | □ |
| 1. Were the exposures measured similarly to assign people to both exposed and unexposed groups? | □ | □ | □ | □ |
| 1. Was the exposure measured in a valid and reliable way? | □ | □ | □ | □ |
| 1. Were confounding factors identified? | □ | □ | □ | □ |
| 1. Were strategies to deal with confounding factors stated? | □ | □ | □ | □ |
| 1. Were the groups/participants free of the outcome at the start of the study (or at the moment of exposure)? | □ | □ | □ | □ |
| 1. Were the outcomes measured in a valid and reliable way? | □ | □ | □ | □ |
| 1. Was the follow up time reported and sufficient to be long enough for outcomes to occur? | □ | □ | □ | □ |
| 1. Was follow up complete, and if not, were the reasons to loss to follow up described and explored? | □ | □ | □ | □ |
| 1. Were strategies to address incomplete follow up utilized? | □ | □ | □ | □ |
| 1. Was appropriate statistical analysis used? | □ | □ | □ | □ |

The Joanna Briggs Institute’s critical appraisal checklist for cohort studies

<https://jbi.global/critical-appraisal-tools>
